# Supplementary material for: α-Synuclein fibril and synaptic vesicle interactions lead to vesicle destruction and increased lipid-associated fibril uptake into iPSC-derived neurons
Source: Commun Biol. 2023 May 15;6:526. doi: 10.1038/s42003-023-04884-1 (PMC10185682; doi:10.1038/s42003-023-04884-1)
Supplement: Supplementary file 2 — Supplementary Information [file 42003_2023_4884_MOESM2_ESM.pdf]

Supporting information

α-synuclein fibril and synaptic vesicle interactions lead to vesicle destruction and increased lipid-associated fibril uptake into iPSC-derived neurons

Amberley D. Stephens, Ana Fernandez Villegas, Chyi Wei Chung, Oliver Vanderpoorten, Dorothea Pinotsi, Ioanna Mela, Edward Ward, Thomas M. McCoy, Robert Cubitt, Alexander F. Routh, Clemens F. Kaminski, Gabriele S. Kaminski Schierle

Contents

Supplementary Figure 1. Representative TEM images show SV range in size from 40-70 nm. 2

Supplementary Figure 2. aSyn fibrils grown in the presence and absence of SV have similar length after sonication. 3

Supplementary Figure 3. Dye only, SV only and dye and SV are barely fluorescent/non-detectable in i3Neurons. 4

Supplementary Figure 4. The proteolysis profile of aSyn fibrils grown in the presence and absence of SV does not differ. 5

Supplementary Figure 5. Model fits to the SANS data at time 0 hrs and 45 hrs using the Guinier-Porod model. 6

Supplementary Figure 6. Single channel images and overlays show SV associated to fibrils after 24 hours. 7

Supplementary Figure 7. STED microscopy and AFM show some fibrils have no SV associated to them after 24 hrs while others are coated in SV. 8

Supplementary Figure 8. Representative TEM images show SV adhering to aSyn fibrils over time and forming a mesh-like structure. 9

Supplementary Figure 9. Monomeric aSyn forms small clusters with SV after 24 hours. 10

Supplementary Figure 10. aSyn and SV incubated alone do not change morphology over 24 hours. 11

Supplementary Figure 11. Correlative STED-AFM shows good correlation, but also presence of ‘dark’ aSyn fibrils. 12

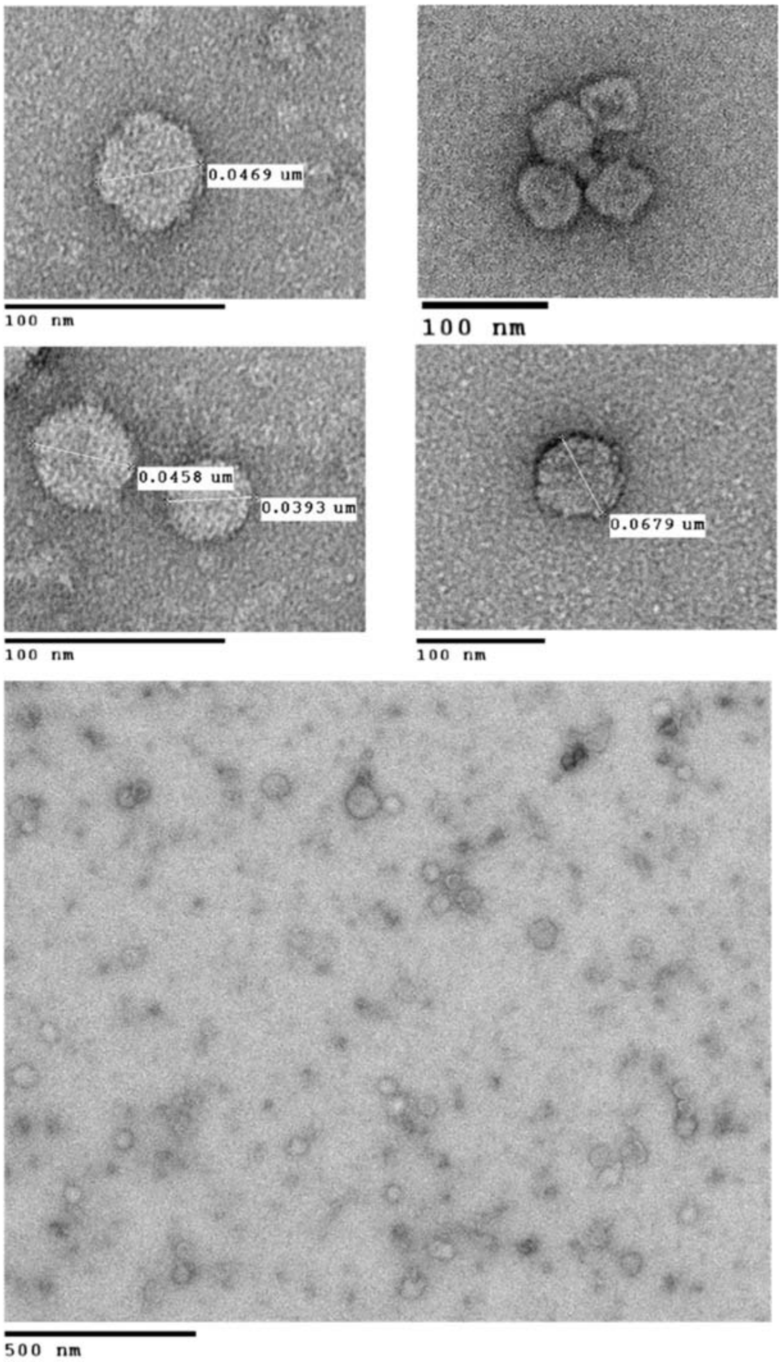

Supplementary Figure 1. Representative TEM images show SV range in size from 40-70 nm. SV were purified from Sprague-Dawley rat brains and imaged using transmission electron microscopy negatively stained with 1% uraynl acetate to determine their size. Point-to-point measurements show the SV to range in size from ~40 nm – 70 nm.

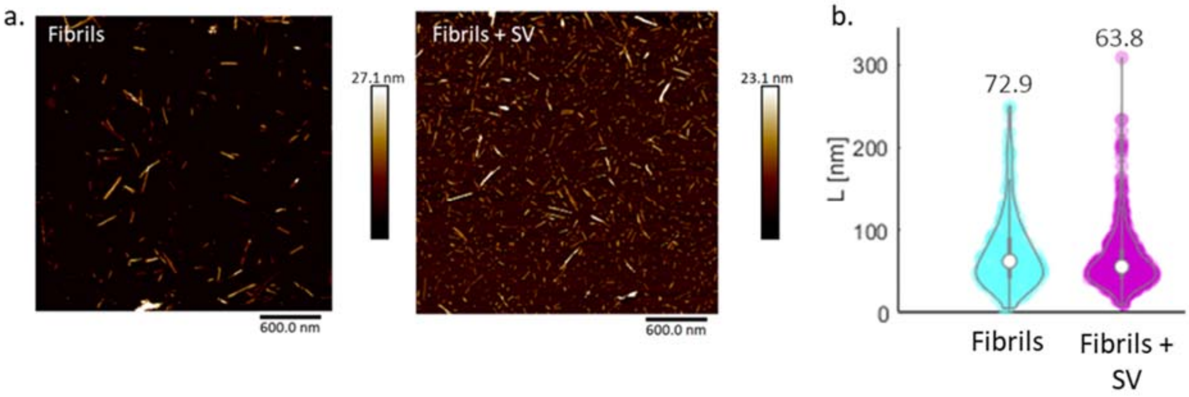

Supplementary Figure 2. aSyn fibrils grown in the presence and absence of SV have similar length after sonication. Monomeric WT aSyn was incubated with 10% dye-labelled aSynC141:AF647N in the absence and presence of SV for one week to form fibrils. The samples were sonicated for 5s at 30% amplitude, diluted to 2.5 µM and incubated on freshly cleaved mica for 20 minutes. The mica was washed three times in dH_2O and AFM images acquired in liquid. a. Representative images are shown for aSyn fibrils only and fibrils + SV. b. Length profiles for fibrils from 11 images of aSyn only fibrils have an average length of 72.9 nm with a s.d. of 45.2 nm (330 fibrils) and 9 images of fibrils + SV have an average length of 63.8 nm with a s.d. of 35.1 nm (600 fibrils).

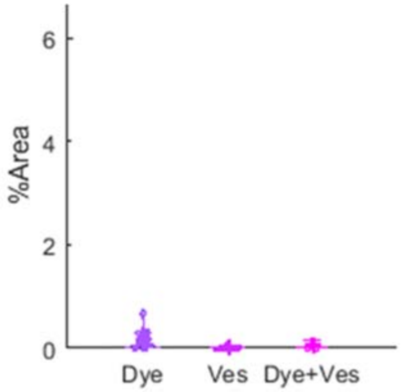

Supplementary Figure 3. Dye only, SV only and dye and SV are barely fluorescent/non-detectable in i3Neurons. To ensure the aSyn and not only the dye was taken into i^3Neurons, the same concentration of free dye, SV only and dye and SV were incubated with i^3Neurons as controls. Minimal fluorescence was observed. Images acquired per sample were Dye = 14, Ves = 14, Dye + Ves = 4

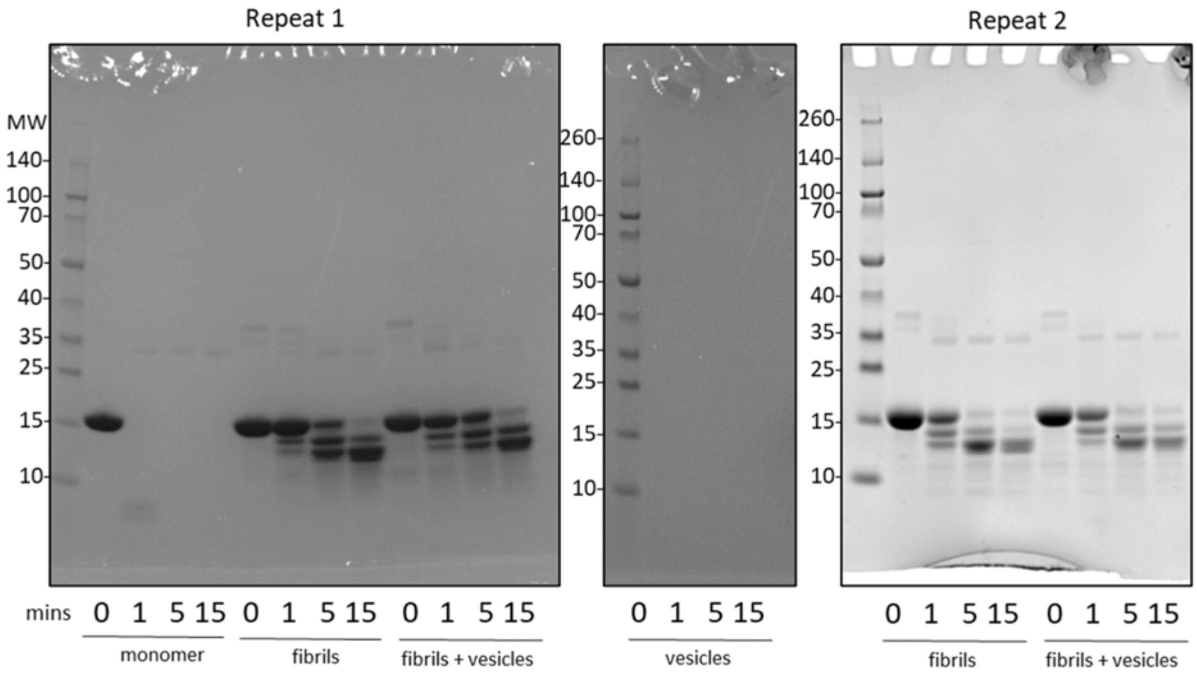

Supplementary Figure 4. The proteolysis profile of aSyn fibrils grown in the presence and absence of SV does not differ. aSyn monomer, SV only, aSyn fibrils and fibrils grown in the presence of SV were incubated with proteinase K for 0, 1, 5 and 15 minutes and the digestion products separated on an SDS-PAGE gel and stained with Coomassie blue. Molecular weight (MW) markers are shown in kDa. Shown are two repeats and the full Coomassie stained blue gels of the digested aSyn samples.

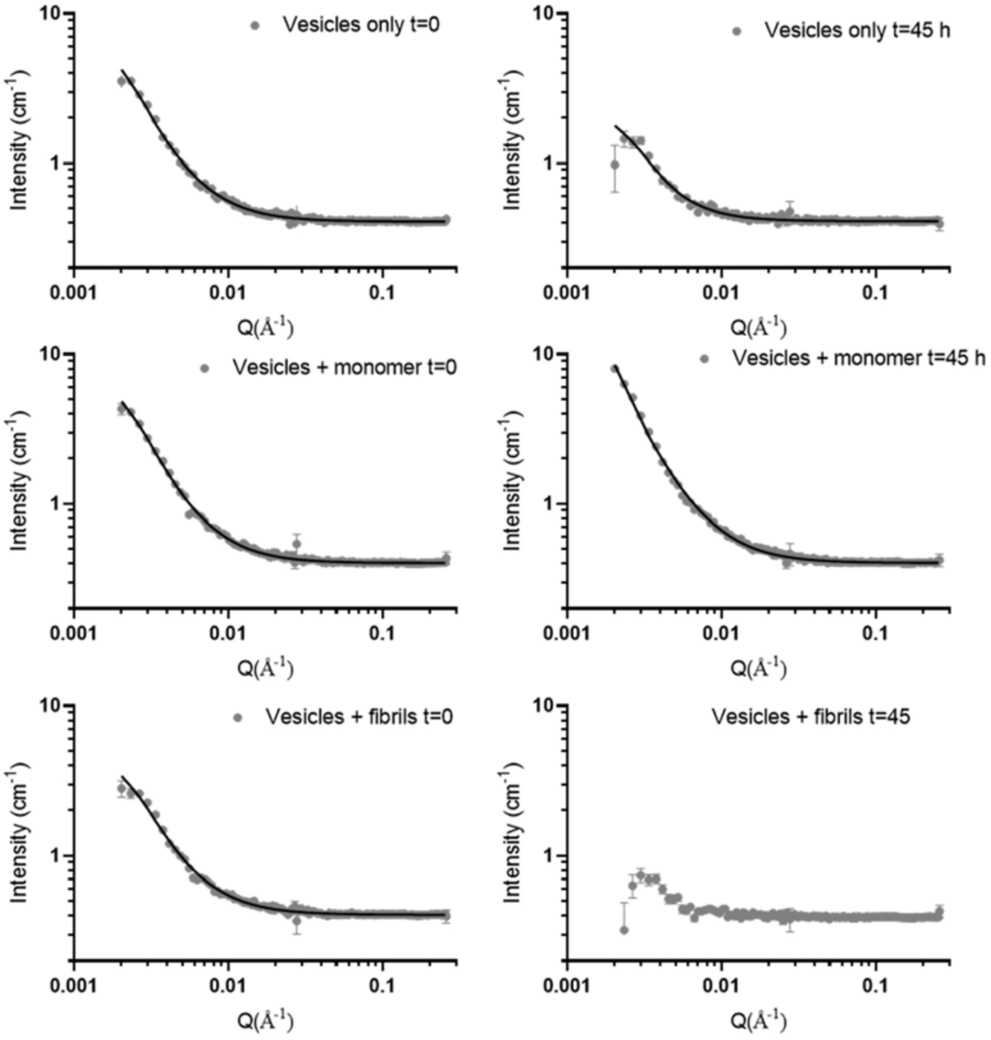

Supplementary Figure 5. Model fits to the SANS data at time 0 hrs and 45 hrs using the Guinier-Porod model. SANS data for SV in 42% D2O with aSyn monomer or fibrils is shown by grey spots. The Guinier-Porod fitting is shown by the black line, apart from for SV + fibrils t=45 where a good fit could not be achieved. Error bars represent s.d.

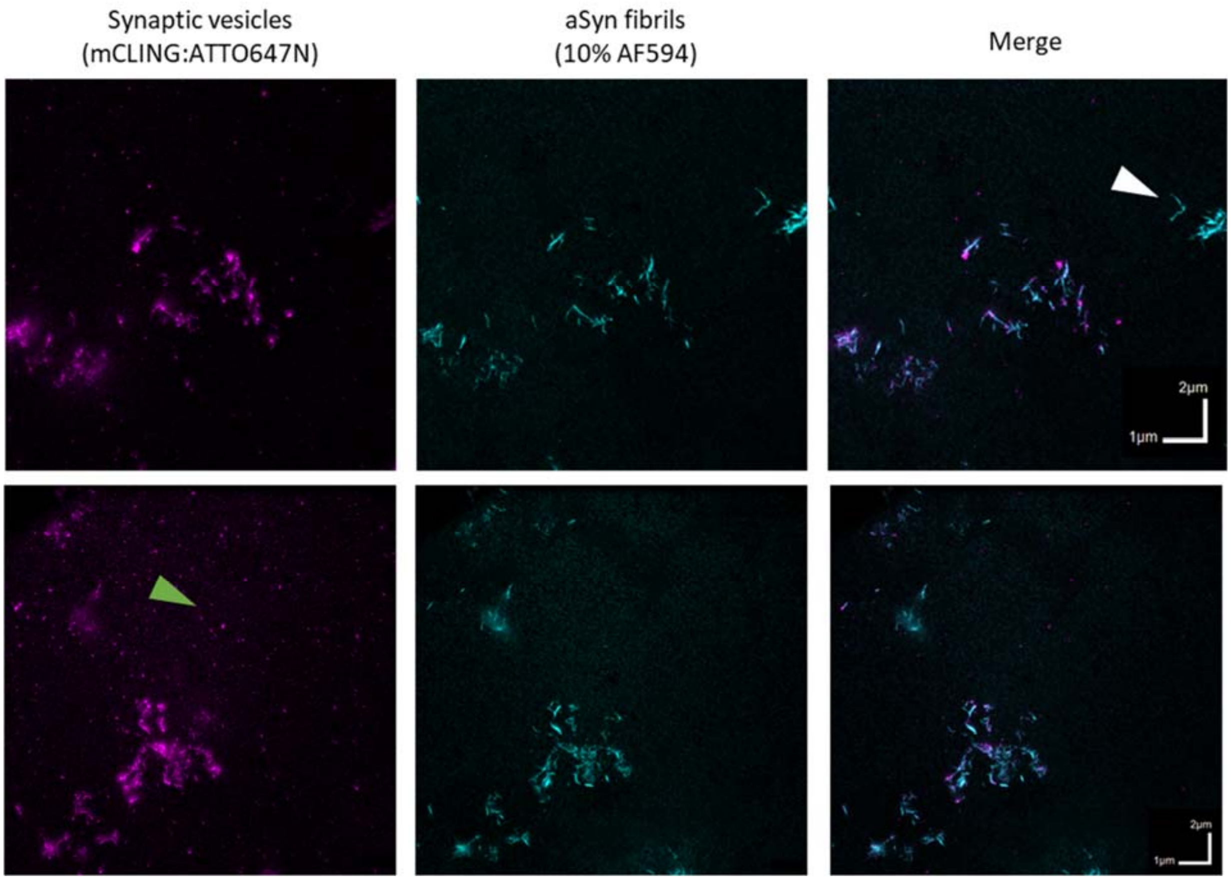

Supplementary Figure 6. Single channel images and overlays show SV associated to fibrils after 24 hours. Representative images show single channels and a merged image for synaptic SV (0.5 mg/mL) stained with a lipid intercalating dye, mCLING:ATTO647N (1:100) (magenta) incubated with 5 µM aSyn fibrils labelled with 10% aSynC141:AF594 (cyan) for 24 hrs. White arrow indicates fibrils with no SV associated. Green arrow indicates SV not associated to fibrils.

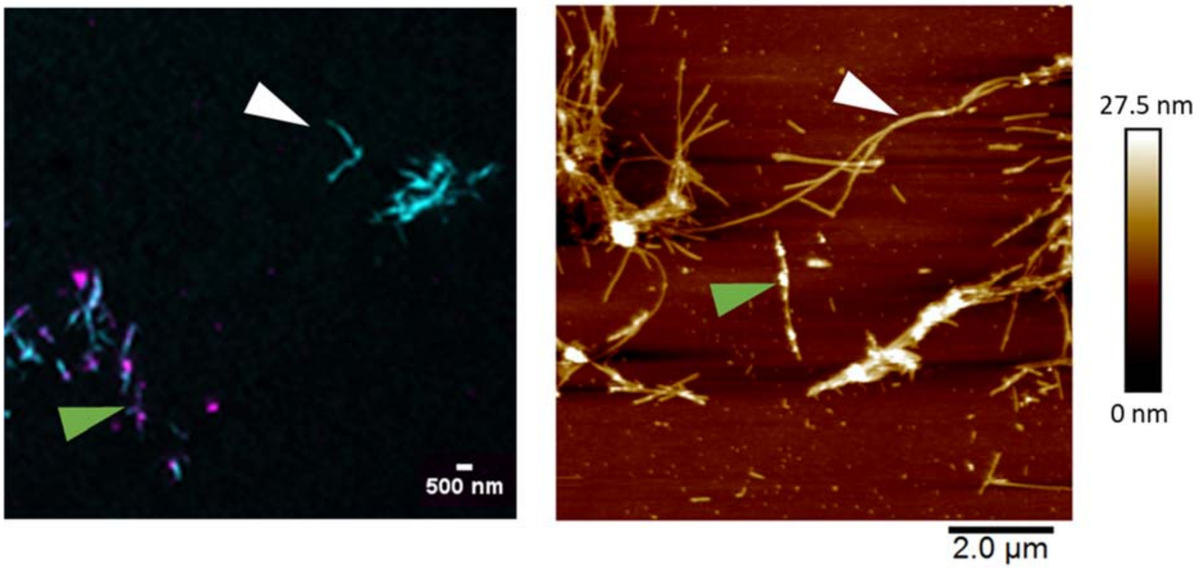

Supplementary Figure 7. STED microscopy and AFM show some fibrils have no SV associated to them after 24 hrs while others are coated in SV. Images acquired after 24 hour incubation of SV (0.5 mg/mL) stained with a lipid intercalating dye, mCLING:ATTO647N (1:100) (magenta) with 5 μM aSyn fibrils labelled with 10% aSynC141:AF594 (cyan). White arrows show fibrils with no SV associated to them in the STED microscopy image (left) and AFM image (right). The green arrows show fibrils coated with SV.

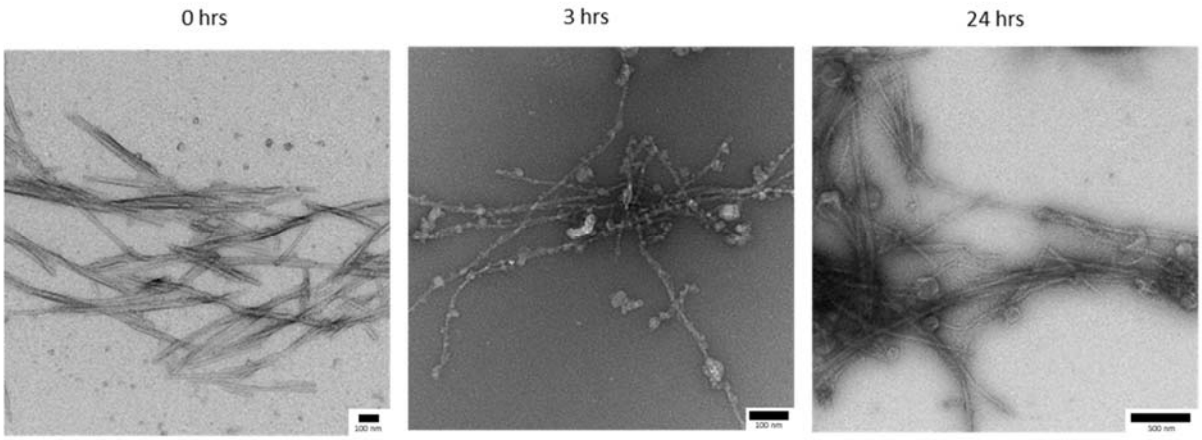

Supplementary Figure 8. Representative TEM images show SV adhering to aSyn fibrils over time and forming a mesh-like structure. SV (0.5 mg/mL) were incubated with 5 µM aSyn fibrils and samples taken for imaging at 0hrs, 3 hrs and 24 hrs. Samples were negatively stained with 1% uraynl acetate. Increasing numbers of SV adhere to the fibrils over time, forming large mesh-like structures of SV and fibrils.

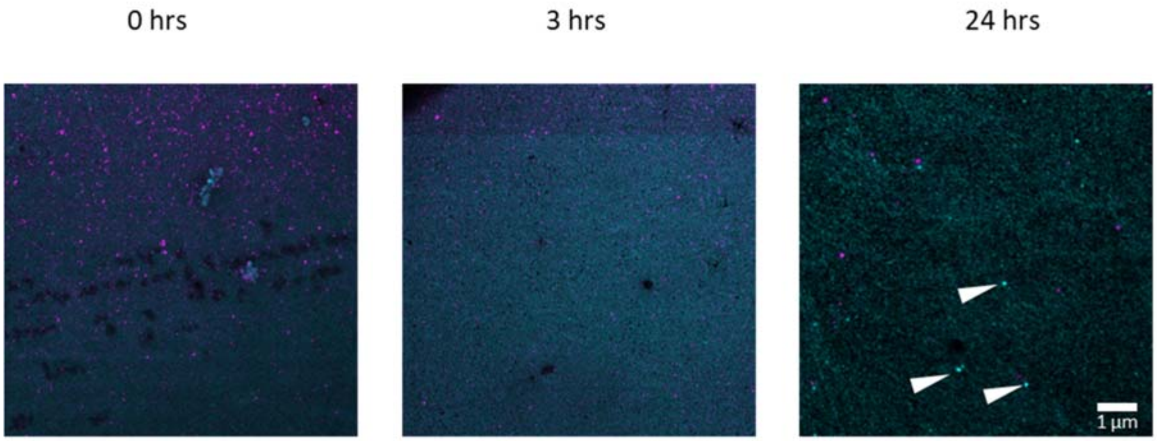

Supplementary Figure 9. Monomeric aSyn forms small clusters with SV after 24 hours. SV (0.5 mg/mL) were incubated with 5 µM aSyn monomer labelled with 10% aSynC141:AF594 (cyan) and incubated at 37°C for 0, 3 and 24 hrs. SV were stained with a lipid intercalating dye, mCLING:ATTO647N (1:100) (magenta). By 24 hours small clusters of aSyn are observed (indicated by white arrows).

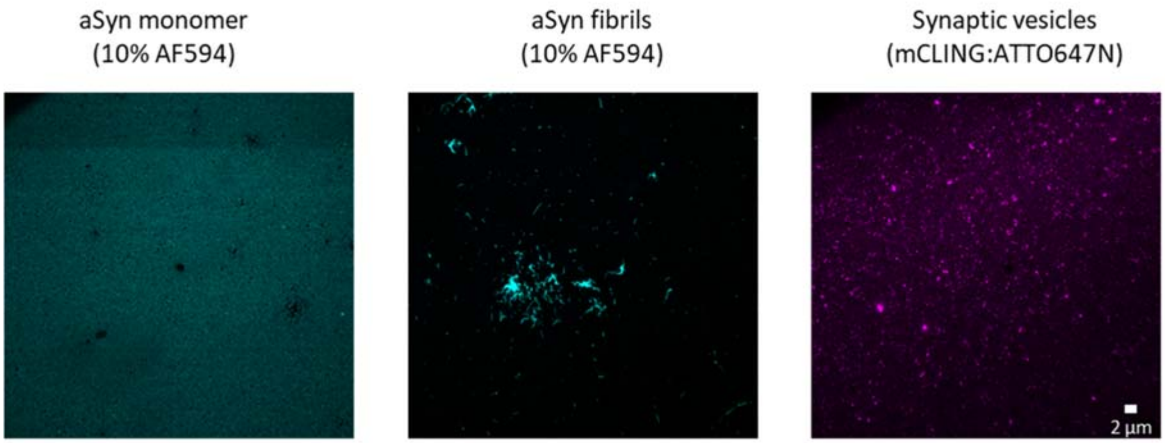

Supplementary Figure 10. aSyn and SV incubated alone do not change morphology over 24 hours. 5 µM aSyn monomer, fibrils labelled with 10% aSynC141:AF594 (cyan) and SV (0.5 mg/mL) stained with a lipid intercalating dye, mCLING:ATTO647N (1:100) (magenta) were incubated individually at 37°C for 24 hrs. There were no drastic changes in morphology compared to time 0 (Figure 1 and Supplementary Figure 3).

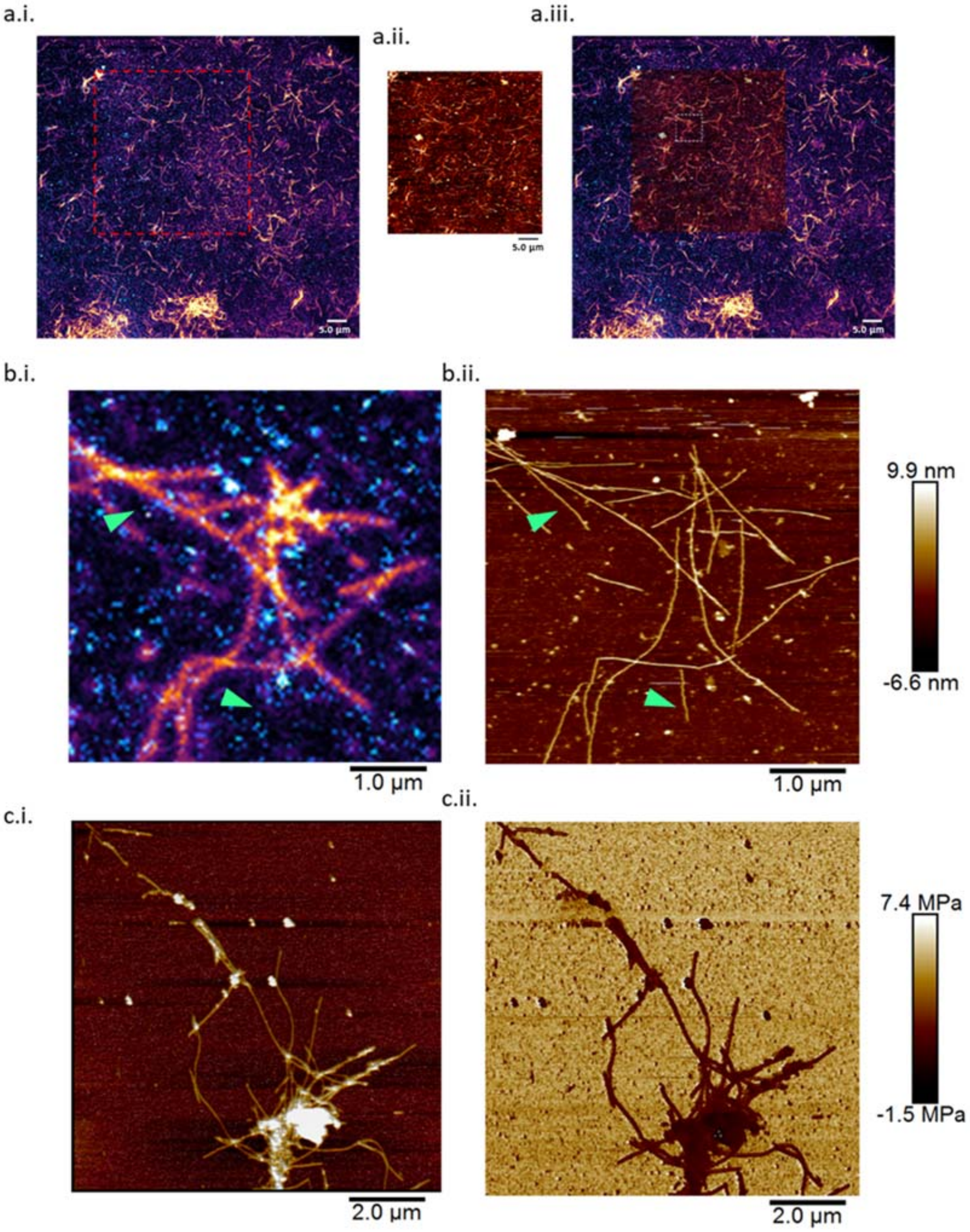

Supplementary Figure 11. Correlative STED-AFM shows good correlation, but also presence of 'dark' aSyn fibrils. (a.i.) STED microscopy image of aSyn fibrils labelled with 10% aSynC141:AF594 (magenta/fire) and SV stained with mCLING:ATTO647N (cyan). A correlative AFM image was then taken within the red dashed box. (a.ii.) The AFM image from within the red dashed box of a.i. (a.iii.) An overlay of the STED microscopy image and AFM image shows good correlation between the fluorescent fibrils and height profile of the fibrils by AFM. (b.i) A zoomed in fluorescence image and correlative (b.ii.) AFM image shows presence of dark' aSyn fibrils (indicated by green arrows) where no fluorescent aSynC141:AF594 was incorporated into the growing fibril. In general the correlation between SV in both images is good. (c.i.) AFM shows SV associating to the fibrils, (c.ii.) The Young's modulus shows the SV are softer than the fibrils.
